# A systematic review of barriers and facilitators for hepatitis B and C screening among migrants in the EU/EEA region

**DOI:** 10.3389/fpubh.2023.1118227

**Published:** 2023-02-15

**Authors:** Chrissy P. B. Moonen, Casper D. J. den Heijer, Nicole H. T. M. Dukers-Muijrers, Ragni van Dreumel, Sabine C. J. Steins, Christian J. P. A. Hoebe

**Affiliations:** ^1^Living Lab Public Health, Department of Sexual Health, Infectious Diseases and Environmental Health, South Limburg Public Health Service, Heerlen, Netherlands; ^2^Department of Social Medicine, Care and Public Health Research Institute (CAPHRI), Maastricht University, Maastricht, Netherlands; ^3^Department of Health Promotion, Care and Public Health Research Institute (CAPHRI), Maastricht University, Maastricht, Netherlands; ^4^Department of Medical Microbiology, Care and Public Health Research Institute (CAPHRI), Maastricht University Medical Centre (MUMC+), Maastricht, Netherlands

**Keywords:** systematic review, hepatitis B, hepatitis C, screening, migrants, facilitators and barriers

## Abstract

**Introduction:**

Hepatitis B and C are a threat to public health. Screening of high-risk groups, such as migrants from high-endemic areas, enables early identification and treatment initiation. This systematic review identified barriers and facilitators for hepatitis B and C screening among migrants in the European Union/European Economic Area (EU/EEA).

**Methods:**

Following PRISMA guidelines, databases PubMed, Embase *via* Ovid, and Cochrane were searched for English articles published between 1 July 2015 and 24 February 2022. Articles were included, not restricted to a specific study design, if they elaborated on HBV or HCV screening in migrant populations from countries outside Western Europe, North America, and Oceania, and residing in EU/EEA countries. Excluded were studies with solely an epidemiological or microbiological focus, including only general populations or non-migrant subgroups, or conducted outside the EU/EEA, without qualitative, quantitative, or mixed methods. Data appraisal, extraction, and quality assessment were conducted and assessed by two reviewers. Barriers and facilitators were categorized into seven levels based on multiple theoretical frameworks and included factors related to guidelines, the individual health professional, the migrant and community, interaction, the organization and economics, the political and legal level, and innovations.

**Results:**

The search strategy yielded 2,115 unique articles of which 68 were included. Major identified barriers and facilitators to the success of screening related to the migrant (knowledge and awareness) and community level (culture, religion, support) and the organizational and economic level (capacity, resources, coordinated structures). Given possible language barriers, language support and migrant sensitivity are indispensable for facilitating interaction. Rapid point-of-care-testing is a promising strategy to lower screening barriers.

**Discussion:**

The inclusion of multiple study designs provided extensive insight into barriers, strategies to lower these barriers, and facilitators to maximize the success of screening. A great variety of factors were revealed on multiple levels, therefore there is no one-size-fits-all approach for screening, and initiatives should be adopted for the targeted group(s), including tailoring to cultural and religious beliefs. We provide a checklist of facilitators and barriers to inform adapted interventions to allow for optimal screening impact.

## 1. Introduction

Hepatitis is a major public health threat calling for global prevention efforts (1). Hepatitis B (HBV) and hepatitis C (HCV) viruses contribute to over 90% of all hepatitis cases, causing an estimated 3 million new infections, 354 million chronic infections, and over 1 million deaths globally in 2019 (2). HBV and HCV infections are often asymptomatic and are usually not discovered until the infection has already progressed into liver disease. Approximately 15–30% of all cases advance into cirrhosis or hepatocellular carcinoma (HCC) (3, 4).

To facilitate hepatitis elimination, the World Health Organization (WHO) outlined the Global Hepatitis Strategy in 2016. This strategy was defined as a 95% and 80% reduction in HBV and HCV, respectively, and a 65% reduction in mortality by 2030, with 2015 as a reference (5, 6). Although this strategy may seem ambitious, adequate implementation of screening, treatment, and (HBV) vaccination can prevent the majority of HBV and HCV-related deaths (5). Especially with the considerable improvement in HCV treatment, opportunities for increasing screening and treatment should be explored (7).

The WHO recommends targeted HBV/HCV testing for high-risk populations, including among others migrants from high-endemic countries, people who inject drugs, men who have sex with men (MSM), and prisoners (8). Targeting specific populations through tailored interventions, also known as micro-elimination, is encouraged by implementation scientists (9). Approximately a quarter of chronic HBV (CHB) and 14% of chronic HCV infections (CHC) in the European Union/European Economic Area (EU/EEA) are attributed to migrants (8). The high burden of HBV and HCV in migrants leads to challenges for both the individuals and the healthcare systems of the host countries, as the health and vaccination status of migrants are often unknown (10, 11). Of the 21 EU/EEA countries that reported on migrant testing policies, only 7 countries had national policies for HBV and 6 for HCV (12). However, the review by Seedat shows high uptake of migrant-targeted screening initiatives in the EU/EEA region (13).

HBV/HCV screening and treatment of migrant populations was estimated as cost-effective in two Dutch studies using a Markov model (14, 15). However, migrant groups can be difficult to reach and may not participate in screening because of experienced barriers in accessing healthcare services (16). To facilitate the uptake of testing, barriers and facilitating factors should be considered when setting up screening initiatives. This systematic review aims to provide an up-to-date overview of barriers and facilitators for HBV and HCV screening among migrants in the EU/EEA to inform the design of interventions to allow for optimal screening potential. The review by Seedat, Hargreaves (13), which identified barriers to and facilitators of hepatitis screening programs in migrants in articles between 1989 and 1 July 2015, was taken as a starting point for the search strategy.

## 2. Methods

### 2.1. Study design and search strategy

In this systematic review, databases PubMed, Embase *via* OVID, and Cochrane were searched for articles written in English published between 1 July 2015 and 24 February 2022. The Boolean search strategy used for our study combined PICO-style keywords for “migrant,” “screening,” and “hepatitis B” or “hepatitis C” and is outlined in Supplementary material 1. Additionally, papers were identified by backward and forward citation searching. The systematic review was conducted in accordance with the Preferred Reporting Items for Systematic Reviews and Meta-Analyses (PRISMA) guidelines (17). There was no funding source for this study.

### 2.2. Study selection

Studies were included if they elaborated on HBV and/or HCV screening in migrant populations, originating from countries outside Western Europe, North America, Australia, or Oceania, and residing in EU/EEA countries or the United Kingdom (UK). The main outcome domains of interest were barriers and facilitators for screening migrants. However, screening studies without these outcome measures were also included if they provided information on strategies to tackle barriers or to facilitate screening participation. Studies were excluded when only general populations (non-migrant) or non-migrant subgroups (e.g., men who have sex with men, sex workers, drug users, and homeless persons) were covered. Studies were also excluded if they did not focus on screening or did not include HBV or HCV. Additionally, exclusion occurred if the study was conducted outside the EU/EEA, did not use a qualitative, quantitative, or mixed-methods design, used a cost-effect methodology, or had adopted a purely epidemiological or microbiological focus, without mentioning potential factors affecting the screening of migrants.

After the removal of duplicates, studies were screened on title and abstract, and irrelevant articles were excluded. Of the remaining articles, the full text was screened. Reviewers RvD and SS assessed the full-article screening individually, blinded to the researcher's assessment. Disagreements were discussed until consensus was reached. In case of disagreement, full-text articles were discussed with senior researchers (CHe, ND, CH) and agreed upon.

### 2.3. Data extraction

The studies' characteristics were extracted and tabulated by design to facilitate comparison. The extracted data were checked for correctness and completeness by RvD and SS. After extraction, the identified barriers and facilitators were categorized according to domains and more specific concepts, inspired by Flottorp, Oxman (18), Grol and Wensing (19), and Fleuren, Paulussen (20). The domains were modified to better match the target group of migrants and recurring concepts in the literature, resulting in seven domains (levels): guideline level, individual health professional level, migrant and community level, migrant and health professional interaction level, organizational and economic level, political and legal level, and innovation level. Concepts were inventoried by domain, resulting in one table for all facilitators and barriers. Perpendicular concepts were reduced based on the definitions of facilitator and barrier applied. Concepts were defined as facilitators if their presence promoted screening and considered barriers if their presence impeded screening. A universal definition of the term “migrant” is lacking (21). Given the often unclear motive for migration. we adhere to the European Union's definition, in which they define migrants as people who change their country of usual residence, irrespective of the reason for migration or legal status (22).

### 2.4. Assessment of risk of bias

The first author assessed the methodological quality of the studies by using the Mixed Methods Appraisal Tool, version 2018 (MMAT) (23). Multiple study designs can be assessed with this tool, as the methodological quality criteria differ for each study design. The judgments of the quality control process were independently verified by reviewers RvD and SS. Divergence between the key author and the reviewers was discussed until consensus was reached.

## 3. Results

### 3.1. Study selection

**Figure 1:**
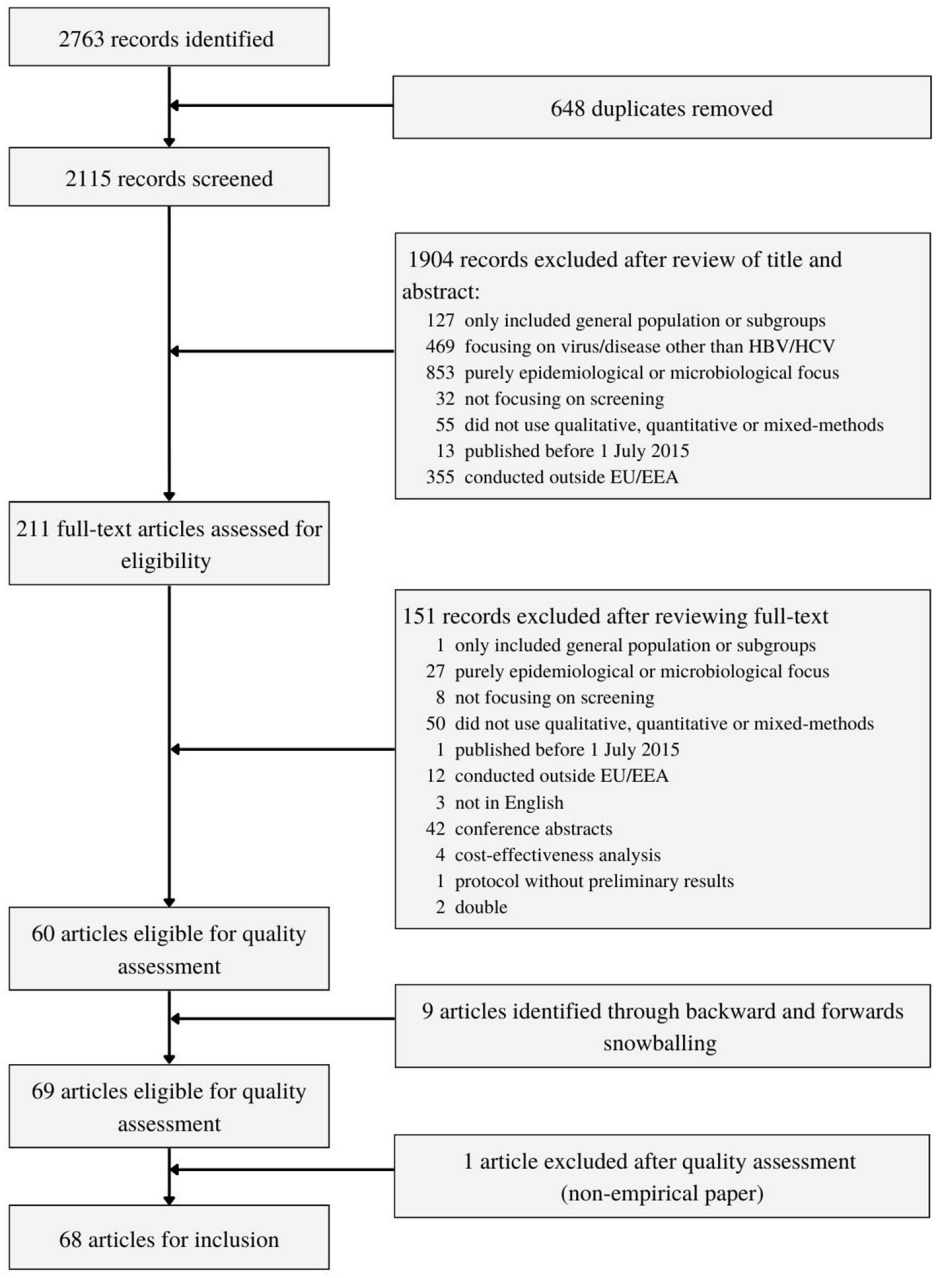
Study selection.

The search strategy yielded 2,763 records (Figure 1). After duplicates were removed, 2,115 unique records were screened by title and abstract. Two hundred and eleven full-text articles were assessed for eligibility, of which 151 were excluded. Additionally, 9 studies were included through backwards and forward snowballing. This resulted in 69 research articles eligible for quality control. One study was excluded after quality control showed it was a non-empirical article. Ultimately, 68 articles were included.

### 3.2. Study characteristics and assessment of risk of bias

Most included studies were observational studies (*n* = 47) (24–70), followed by qualitative studies (*n* = 13) (71–83), experimental studies (*n* = 7) (84–90), and mixed-method studies (n=1) (91). All the studies were finished, except for the study of Thonon, Fahmi (90). Full details of the study characteristics are outlined in Supplementary material 2. Overall, quality criteria were reported adequately in the included studies (Supplementary material 3). However, the risk of non-response bias was not often discussed and was therefore difficult to assess. The identified facilitators and barriers are tabulated in Table 1 and major concepts are discussed below.

**Table 1:** Barriers and facilitators for HBV/HCV screening among migrants.

**Level**	**Facilitators**	**Barriers**
Guideline level	Presence of guidelines Awareness of guidelines Adherence to guidelines Exceeding restricting guidelines	
Individual health professional level	Knowledge/awareness Skills Proactive approach	Competing patient health priorities
Migrant and community level	**Migrant** Knowledge/awareness Risk perception Perceived severity Perception of healthcare needs Self-efficacy Perceived personal benefit Positive attitude (toward prevention)	**Migrant** Misconceptions Logistical barriers (time, transport and money) Fear Competing priorities
	**Community** Religion Social influence/support Community protection Engagement of community-based organizations Engagement of key figures	**Community** Fatalism Social influence/fear of rejection Threat to fulfilling social roles Stigma Cultural barrier Disadvantaged social position
Interaction between migrant and health professional level	Language support Cultural competence Migrant sensitivity Trust Comprehensible information Staff's (positive) attitude and behavior Time for patient Healthcare navigation Positive previous care experience Training of staff in using an interpreter	Unfamiliarity with healthcare Illiteracy Language barrier Cultural barrier
Organizational and economic level	Capacity of staff Training of staff Available resources Efficient care offer Financial arrangements Coordinated healthcare structures Use of protocols (systematic approach) Proper data management Accessible screening Active recruitment Collaboration with authorities or (local) institutions Dedicated services Comprehensive prevention approach Anonymous testing/ensuring privacy Outreach and screening in the community Informative activities Financial incentives	Long waiting times High workload of staff Uncomfortable surroundings Logistical barriers
Political and legal level	Healthcare access entitlement Enabling regulations, rules, and policies (International) data management (Perceived) obligation Political awareness and prioritization Legal support National efforts/coordination Engagement of advocates	Fear of deportation Bureaucratic barriers Regional differences Migrant mobility
Innovation level	Innovation in diagnostics In accordance with patients' preferences In accordance with professionals' preferences Advantages in practice	Insufficient accessibility (for low computer literacy) Insufficient (perceived) accuracy (sensitivity/specificity) Insufficient feasibility (test failures, lack of trained staff)

### 3.3. Barriers and facilitators per level

#### 3.3.1. Guideline level

Guidelines, such as WHO, Centers for Disease Control and Prevention (CDC) and National Institute for Health and Care Excellence (NICE) guidelines are empirically based and therefore a good starting point for designing and implementing screening initiatives, as supported by Norman, Comeche (55). However, studies did not often mention whether and on what guidelines screening was based. If standardized guidelines for screening, referral and treatment are lacking, chances of missed infections and undesirable variations in practice increase (74). Despite the existence of guidelines, professionals may not be aware of their existence or they may not adhere accordingly (40, 67). This is highlighted in the study of Evlampidou, Hickman (40), in which 14 out of 15 general practitioners (GPs) were unaware of the NICE guidelines recommending routine HBV testing in migrants. The disease prevalence and risk factors in the home country of migrants can be an indicator for targeted screening, as pointed out by among others Donisi, Gerna (38).

#### 3.3.2. Individual health professional level

To be able to convey the importance and motivate the target group to be screened for HBV/HCV, professionals must be well aware and knowledgeable about these viruses and indicators for testing (40, 60). Competing patient health priorities, for example, diabetes or cardiovascular disease, may be prioritized by health professionals over asymptomatic HBV/HCV screening (70). As shown by for example Andersen, Kruse (24) and Kloning, Nowotny (49), screening should be performed by experienced and specialized doctors with adequate medical education and skills regarding the disease and the target group. Furthermore, a proactive approach of professionals is desirable because this facilitates timely linkage to care and vaccination (54). In contrast, a low alerting role and low motivation of health professionals can hinder the identification and uptake of screening (47).

#### 3.3.3. Migrant and community level

Given the interconnectedness of the community in the lives of migrants, the community and the migrant cannot be viewed completely separated and are therefore grouped into one domain. At the migrant level, knowledge and awareness were often inseparably linked and are major concepts to consider. In comparison to other infectious diseases, human immunodeficiency virus (HIV) for example, hepatitis is relatively unknown with various misconceptions about its nature, transmission and health risks (68, 71, 73). For example, in the study of Cochrane, Collins (73) hepatitis was mistaken for jaundice and the asymptomatic course was often unknown. The lack of symptoms is an important barrier to screening, as shown by Hamdiui, Steenbergen (42). Filling knowledge and awareness gaps through education, can increase the perceived risk, perceived disease severity and perceived healthcare needs, which are predictors for screening (41, 43, 73, 77).

Another recurring concept was self-efficacy—the belief in one's ability to engage with screening (43, 78). As shown by Hamdiui, Stein (43), self-efficacy is an important predictor of screening participation. Empowerment strategies, for example by education, can be deployed to increase self-efficacy (87).

Furthermore, the perceived outcome expectancy can influence the willingness to participate in screening. Screening can provide the individual with a personal benefit, such as clarity on infection status or treatment in case of infection (43, 71). Nevertheless, low perceived personal benefit or low motivation can impede screening willingness (90). Additionally, the perceived burden of screening may be a determining factor for screening participation (43). Logistical barriers such as finite financial resources, lack of time and geographic barriers, including transport and lack of locally available services, can be decisive (60). Also, other pressing matters, such as administrative procedures for approval of a residence permit, may require priority (58, 71). The disadvantaged social position of migrants and socio-economic insecurity can be barriers to screening (61, 83).

Importantly, migrants should be approached sensitively (53). We recognize that migrants often flee from war or misery, often accompanied by negative experiences, such as the loss of loved ones and even torture and sexual abuse (62, 66). The intense migration journey may lead to trauma and fear. Also, people may have anxiety about the screening itself, including fear of drawing blood, anxiety about receiving the test results, and fear about disclosure of the outcome (71, 75, 78).

At the community level, a strong sense of community and group identity often prevail in migrant groups (78). Next to protecting one's own health, screening for infectious diseases is a way to protect the community by preventing transmission (82). Moreover, the social environment is an important influence, as participation can depend on the opinion and support of the community (71). Although the community can be perceived as a source of support, fear of rejection by the community in case of infection may negatively influence screening participation (78). Furthermore, social roles, which are behaviors expected as a member of a group, can encourage screening participation through the experienced duty to look after ones family (73). However, the fear of testing positive and not being able to fulfill social roles can be a barrier to screening. (78).

Likewise, religion can be an important driver of whether or not to participate in screening. Screening can be perceived as a duty of faith to look after one's own health (77, 80). On the other hand, diseases such as hepatitis can be seen as events beyond ones control, also known as fatalism (30, 78). To increase the reach of screening, religious key figures can be deployed to further disseminate screening aims and importance (29). For example, imams (mosque leaders) can spread knowledge about the purpose of screening during mosque attendance (47). Community stakeholders, such as councilors, can also be involved to raise knowledge and (political) awareness (47). To increase input from migrants in screening design and to optimize migrant sensitivity, community-based organizations can be engaged. Aside from increasing the reach of screening, the engagement of community stakeholders may decrease feelings of shame (43).

Negative attitudes or behaviors toward someone who tests positive for HBV or HCV, also known as stigma, was another recurring concept. On the one hand, hepatitis can be associated with drug use and risky sexual behavior, which can lead to shame about discussing hepatitis and getting tested (80). On the other hand, there may be less stigma because of gaps in knowledge about transmission routes. For example, in the study of Azadi, Dollat (71), hepatitis was regarded more as a medical problem like diabetes and less as a sexually transmitted disease (STI), making testing less emotionally charged than testing for highly stigmatized infections such as HIV. Furthermore, migrants may feel stigmatized if a single migrant group is invited for screening. To avoid stigmatization, screening can target migrant groups from multiple continents (71, 73).

#### 3.3.4. Interaction between migrant and health professional level

How information about screening is conveyed is important, especially given the possible language barrier migrants may experience. To make an informed decision about screening participation, migrants should be provided with clear and comprehensible information (77). Information was provided in almost all studies, including translated informational materials, personal contact with a health professional, and educational films.

Grasping the information can be impaired by limited discussion and communication issues due to language barriers and cultural differences (57). As portrayed by Nkulu Kalengayi, Hurtig (79), the majority of invited migrants did not show up at their screening appointment, as the invitation letters for the screening were only in Swedish. In case of language barriers, it is desirable to offer language support by providing translated information, without the use of jargon, taking into account different levels of (health) illiteracy (75, 76, 80).

Besides translated materials, language support can be provided by (adequate) interpreting (60, 81). As was done in many studies, the interpretation can best be facilitated by a cultural mediator, a person who mediates both linguistically and culturally. Cultural mediators enable meaningful information exchange and facilitate the understanding of needs (54, 71). Furthermore, cultural mediators can assist in establishing trust relationship (71). (Cultural) mistrust may be related to skepticism about how authorities operate, mistrust in western medicine and practices, and lack of confidence in (personal) data storage (74, 76, 81). Other potential cultural barriers should be taken into account, such as discomfort about the sex of a physician or joint educational meetings (80, 81).

Culturally competent and migrant-sensitive health professionals should adopt an appropriate counseling approach. Although this seems obvious, health professionals should be friendly; showing a positive attitude and behavior without prejudice and discrimination, and taking sufficient time for the patient (37, 60, 76). This approach should be respectful and holistic, considering complex (healthcare) needs and possible trauma given possible negative experiences during the migration journey (79). The experience of (previous) care moments and the (dis)satisfaction of needs can determine trust and influence participation in screening (78, 79). Next to proper treatment of migrants, professionals should be mindful of the range of awareness, knowledge, and emotions (37, 76). In case of unfamiliarity with the healthcare system, a (peer) navigator can help with setting up health insurance and linkage to care and follow-up of patients (58).

#### 3.3.5. Organizational and economic level

For screening to be effective, a comprehensive prevention approach should be offered that includes screening, linkage to care and treatment, and ideally also source and contact tracing and vaccination (29, 46). When comparing screening initiatives with high participation rates (>95%), we see that all studies refer positively tested patients to care (27, 31, 37, 44, 46, 50, 58, 59). Other noteworthy similarities were the systematic approaches to multi-infectious disease screening, using appropriate procedures and forms, carried out by skilled personnel. Testing for multiple diseases is efficient as it saves time, costs, and reduces the burden for both the patient and the healthcare system. Barriers related to financial resources were often met by financial support from the government, minimizing financial barriers for the patients.

Furthermore, screening should be accessible, for example by offering screening by clinics with flexible opening hours or locally available dedicated services (60, 81). Long waiting times and other service issues, can be hindering patients' engagement in screening. Logistical barriers can be reduced by outreach and screening in the community itself (50, 58, 86).

Screening should be a streamlined and efficient care offer. This can be realized by coordinated healthcare structures, multidisciplinary teamwork and collaboration with authorities or (local) institutions (39, 51). Sufficient capacity is essential, considering high workload is a major factor for low enrolment, according to the study of Zampino, Capoluongo (70). A facilitator for screening mentioned by health professionals was incentives for testing (60), which was shown (cost)effective in experimental studies (85, 89).

Organizations should carefully consider their data management. Routine recording of data by standardized screening questionnaires and dedicated databases is recommended (68). However, the privacy of the migrants should always be warranted. The possible experienced threshold for screening can be lowered by ensuring the privacy of the patient by testing anonymously, as was done, for example, in the study of Coppola, Monari (33).

#### 3.3.6. Political and legal level

Political awareness is desirable for the prioritization of HBV and HCV. Engaging advocates can increase this political awareness by pleading the importance of the issue to (local) decision-makers (81). Without awareness and prioritization, national and local efforts and coordination will be sparse (81).

Free, easy and full access to healthcare services benefits screening, as seen in the study of Salas-Coronas, Cabezas-Fernández (64). Rules, regulations and policies can both hinder or facilitate healthcare access. For example, in Germany, the special needs of unaccompanied minors (UAMs) are defined in the Youth Welfare act and the UAMs receive more assistance in accessing healthcare (52). Legal support by, for example, a social worker, may further facilitate the comprehensibility of rights and healthcare access by for example assisting with the burdensome and time-consuming bureaucratic processes (25).

Regulations can also be hindering as they may limit healthcare access. For example, in some countries having a residence permit may be a prerequisite for health insurance (83). Furthermore, in the studies with high participation rates, screening took place most often in reception or refugee centers (27, 37, 44, 59). The high participation rate in these settings may be influenced by fear of deportation because incoming migrants might think screening is an obligatory element of the asylum application. Clear communication at the political and legal level about the independence of the outcome of the screening on the residence permit, can address this (71).

High migrant mobility, for example, due to the allocation of refugees, complicates the communication of results and follow-up (25). A dedicated national network to monitor treatment can contribute to the completion of the recovery process (54). Moreover, (inter)national data management using patient numbers can contribute to better care delivery and avoid the unnecessary burden and additional costs of screening, as suggested by healthcare professionals involved in Swedish screening (74).

#### 3.3.7. Innovation level

In recent studies, promising innovations for screening are emerging. One such innovation is point-of-care testing (POCT), which involves near-patient diagnostic testing and analysis, outside of a laboratory. The rapid results save time and money and address geographical problems and thus improve the cascade of care (86). Infected patients can be linked to care more quickly without losing patients to follow-up in a second visit—resulting in a higher chance of treatment and recovery. POCT can also advance healthcare during epidemics such as COVID-19, as this testing can be deployed anywhere with minimum skilled personnel (58).

Patients often prefer rapid testing because it is less stressful and more practical than standard testing (84). POCT is also accepted by professionals, as it simplifies consultation and it is easy to incorporate into the routine workflow (88). However, POCT can be less feasible due to finger prick failure (88). In addition, POCT can be (perceived as) less reliable than laboratory diagnostics and patients can experience discomfort by not feeling prepared for the test (75). A different method, the dried bloodspot (DBS) method, also makes blood collection by a fingerpick possible. However, the blood sample needs to be analyzed in a laboratory, making the turnover less efficient than POCT (58).

Other innovations were web and mobile applications, such as the multi-lingual application “RiskRadar” which uses a risk calculator to support prevention, testing and linkage to care of infectious diseases and STIs (87). However, the potential accessibility of “RiskRadar” was most likely sub-optimal due to the COVID-19 pandemic and limited computer literacy. Furthermore, Sequeira-Aymar, Cruz (89) showed that a digital risk assessment tool in primary care for individual screening criteria can improve the number of diagnoses. Thonon, Fahmi (90) are endeavoring to bridge language barriers between the patient and healthcare professionals by using the Apidé app. This app will help with screening for HBV, HCV and HIV among migrants with limited French-speaking skills.

## 4. Discussion

This systematic review identified barriers and facilitating factors for HBV and HCV screening in migrants in the EU/EEA region. Many of the here identified factors were in line with previous review-studies examining infectious disease screening initiatives among migrants (13, 16, 92, 93). By discussing concepts according to seven domains inspired by Flottorp, Oxman (19), Grol and Wensing (20), and Fleuren, Paulussen (21), we allowed the inclusion of less frequently featured domains, such as healthcare innovations, since in recent years more innovations are being applied in screening, such as rapid POCT (84, 86, 88).

This review showed that many screening initiatives for HBV, HCV and other infectious diseases among migrants have been implemented, following the WHO call for global action (5). Studying these initiatives showed that screening often involved a comprehensive prevention approach, including information provision, (free) diagnostics, linkage to care of patients and occasionally vaccination and source and contact tracing. We share the vision of Noori, Hargreaves (94) of universal accessible healthcare access, including free, voluntary and non-stigmatizing screening with appropriate linkage to care. Linkage to care is an important aspect of screening since treatment initiation is a prerequisite for screening to be (cost)effective (15). In the systematic review of Seedat, Hargreaves (13), single infectious disease screening was prevailing in previous years. Now, the shift to multi-infectious disease screening took place, as screening for multiple-infectious diseases dominated the more recent literature, which is consistent with WHO recommendations (95).

Most of the here identified barriers and facilitators related to the individual migrant and community level, as well as to the organizational and economic level. A key concept was the lack of knowledge and awareness of HBV/HCV within migrant groups, leaving many unaware of their risk (42). Lack of knowledge was also a featured concept in other systematic reviews (92, 96). Since there can be a stigma surrounding these infections, information should be carefully compiled (97).

Additionally, the studies often highlighted language and cultural barriers, consistent with an umbrella review of barriers for migrants in accessing health care (98). As language differences can hinder informed decision-making and screening uptake, language support was provided in most initiatives by interpreters and cultural mediators, and translated materials. However, the terms interpreter and cultural mediator were used interchangeably, and little was elaborated on the training, as was also raised by McGarry, Hannigan (99). Other systematic reviews also emphasize the importance of migrant-sensitive and culturally competent screening initiatives (13, 92). Besides cultural mediators, key figures, for example, imams and community-based organizations, can contribute to designing migrant-sensitive initiatives. Furthermore, they enable the dissemination of knowledge and awareness, and stigma reduction in the target group (100). Also, the method respondent-driven sampling (RDS) can help with recruitment, given the close ties within the community. Although often labor-intensive given that support is often requested, this method can be deployed (additionally) as it enables recruitment through the social network of a sample (42, 101).

Patients do not always finalize treatment (13). An electronic patient file with (internationally) allocated patient numbers could increase the interchangeability of health, infection, vaccination and treatment status when entering a different (part of a) country (67, 74). This can prevent unnecessary screening and thus decreasing the burden on migrants and saving costs. In case of treatment, an interchangeable patient number can reduce the chances of losing sight of the patient and not completing treatment. However, the utilization of electronic patient numbers requires tremendous coordination and cooperation between countries, including privacy and data security concerns.

### 4.1. Strengths and limitations

This review has several strengths and limitations. Multiple study designs were explored to accommodate a broad inventory of barriers and facilitators for screening. This provided a comprehensive overview of the identified concepts influencing HBV/HCV screening in migrants. Two reviewers independently assessed the full-article screening (blinded from the first author), the quality assessment and the data abstraction. Although observational studies mainly focused on finding markers for (hepatitis) disease, important lessons can be learned by exploring the methodological elements of these studies. However, the effectiveness of these methods is hard to determine, making it difficult to value the power of these elements. Nevertheless, to put some value to this, the participation rate of the screening studies was taken into account.

A key limitation of the evidence was that observational screening studies elaborate little on the methodology, acceptability, participation rate, reasons for non-participation, and lessons learned. Limited information regarding these important elements hinders mutual learning. Furthermore, the search strategy was limited to all English articles due to lack of time and resources to include other languages. Given that only studies among migrants within the EU/EEA region were examined, this study has limited generalizability.

### 4.2. Implications

In designing and implementing HBV/HCV screening in migrant populations, barriers should be addressed and facilitating factors should be featured. However, there is no one-size-fits-all approach to screening; tailoring to the specific target group(s) is required. Further consideration should be given to the heterogeneity of migrant groups, including cultural and religious beliefs. To keep the burden for the migrants, organization and staff as low as possible, screening initiatives must be efficient by reduced moments of care, structured processes and streamlined pathways.

As for policy, there should be global, European and national attention to HBV and HCV. Screening is often set up on a project basis, with a brief focus on HBV/HCV. However, to achieve long-term health benefits in migrant groups, screening should be a continuous and integrated process. To this end, it is important to clearly define who is responsible for conducting the screening. A clear path from start to finish of screening including predefined guidelines, protocols, and working arrangements avoids variations in practice and infections being missed and untreated.

Further research is needed to identify appropriate screening methodologies and to explore migrant perspectives. To gain insight in what works and what does not regarding screening, authors should elaborate on the lessons learned. Innovations such as POCT, and applications for identification of high-risk individuals and translation can eliminate barriers and can improve delivering screening and adherence to treatment (84–90). While promising, these innovations need to be adequately tested in validation studies in different target populations.

### 4.3. Conclusion

In the EU/EEA region, migrants account for a large share of the infectious disease burden. To protect the host country and to address individual healthcare needs, screening initiatives for identifying HBV and HCV infections and complementary linkage to care should be initiated. To keep the burden as low as possible for the patient, organization and staff, it is important that screening initiatives are organized efficiently by structured processes and streamlined pathways. This systematic review provides a synopsis of recent literature regarding barriers and facilitators in HBV/HCV screening in migrants and can be used as a checklist to design a screening program in practice. Addressing these barriers and implementing these facilitators for this target group can facilitate the establishment of a sophisticated and optimized screening interventions.

## Author contributions

The search strategy was set up by CM, with guidance of supervisors CHe, ND, and CHo. CM conducted the inclusion and exclusion process, the quality assessment, and the data extraction. RD and SS blindly and independently appraised all assessments of CM. Differing assessments were discussed between CM, RD, and SS or with supervisors CHe, ND, and CHo, depending on the certainty of the verdict. CM wrote the original draft. Guidance was provided throughout the entire process by supervisors CHe (first supervisor), ND, and CHo. All authors contributed to reviewing and editing of the draft. All authors had full access to all the data in the study and had final responsibility for the decision to submit for publication.

## Supplementary material

Supplementary material 1 Search strings.

Supplementary material 2 Study characteristics by design.

Supplementary material 3 Quality Assessment MMAT.
