# Supplementary material for: A systematic review of barriers and facilitators for hepatitis B and C screening among migrants in the EU/EEA region
Source: Front Public Health. 2023 Feb 15;11:1118227. doi: 10.3389/fpubh.2023.1118227 (PMC9975596; doi:10.3389/fpubh.2023.1118227)
Supplement: Supplementary material 1 — Search strings. [file Table_1.DOCX]

Supplementary Material

**A systematic review of barriers and facilitators for hepatitis B and C screening among migrants in the EU/EEA region**

**Chrissy P.B. Moonen^*^, Casper D.J. den Heijer, Nicole H.T.M. Dukers-Muijrers, Ragni van Dreumel, Sabine C.J. Steins, Christian J.P.A. Hoebe.**

*** Correspondence:** Chrissy Pierre Brigitte Moonen: [chrissy.moonen@maastrichtuniversity.nl](mailto:chrissy.moonen@maastrichtuniversity.nl)

**Supplementary Material 1. Search strings**

***Database: PubMed***Last searched for: 02-02-2022
Custom range: from 2015-current, English, Dutch
Results: 835 results

Search string: ((((Hepatitis OR Hepatitis B OR Hepatitis C OR HBV OR HCV OR Viral Hepatitis OR Hepatitis B virus OR Hepatitis C virus OR Hepatitis B, chronic OR Hepatitis C, chronic) AND (Screening OR Diagnostic test OR infection control OR Screen OR Test OR Detect OR Screening program OR Secondary Prevention) AND (Migrants OR Migrant OR Immigrant OR Immigrants OR Migration OR Immigration OR Settler OR Incomer OR Foreign born OR Foreign-born OR First-generation immigrant OR FGI))))

***Database:*** ***Cochrane***Last searched for: 14-02-2022
Custom Range: From 01/07/2015 to 14/02/2022:
Results: 44 results (18 Cochrane reviews, 2 Cochrane protocols, 24 trials)

Search string:
ID Search
#1 MeSH descriptor: [Transients and Migrants] this term only
#2 ''Migrants''
#3 ''Migrant''
#4 ''Immigrants''
#5 ''Immigrant''
#6 ''Settler''
#7 ''Incomer''
#8 ''Foreign born''
#9 ''Foreign-born''
#10 ''First-generation immigrant''
#11 ''FGI''
#12 #1 OR #2 OR #3 OR #4 OR #5 OR #6 OR #7 OR #8 OR #9 OR #10 OR #11
#13 MeSH descriptor: [Mass Screening] explode all trees
#14 ''Screening''
#15 ''Diagnostic test''
#16 ''Screening test''
#17 MeSH descriptor: [Infection Control] this term only
#18 ''Infection control''
#19 ''Screen''
#20 ''Test''
#21 ''Detect''
#22 ''Screening program''
#23 MeSH descriptor: [Secondary Prevention] this term only
#24 ''Secondary prevention''
#25 #13 OR #14 OR #15 OR #16 OR #17 OR #18 OR #19 OR #20 OR #21 OR #22 OR #23 OR #24
#26 MeSH descriptor: [Hepatitis] this term only
#27 ''Hepatitis''
#28 MeSH descriptor: [Hepatitis B] this term only
#29 ''Hepatitis B''
#30 MeSH descriptor: [Hepatitis C] this term only
#31 ''Hepatitis C''
#32 ''HBV''
#33 ''HCV''
#34 ''Viral Hepatitis''
#35 MeSH descriptor: [Hepacivirus] this term only
#36 ''Hepatitis C Virus''
#37 MeSH descriptor: [Hepatitis B virus] this term only
#38 ''Hepatitis B Virus''
#39 MeSH descriptor: [Hepatitis B, Chronic] this term only
#40 ''Hepatitis B, chronic''
#41 MeSH descriptor: [Hepatitis C, Chronic] this term only
#42 ''Hepatitis C, chronic''
#43 #26 OR #27 OR #28 OR #29 OR #30 OR #31 OR #32 OR #33 OR #34 OR #35 OR #36 OR #37 OR #38 OR #39 OR #40 OR #41 OR #42
#44 #12 AND #25 AND #43

***Database: Embase via Ovid***Last searched for: 24-02-2022
Custom range: Year 2015-current
Results: 1883 text results

Search string: (hepatitis or hepatitis B* or hepatitis C* or HBV* or HCV* or viral hepatitis or hepatitis B, chronic OR hepatitis C, chronic) and (screen* or diagnos* or infection control or test* or detect* or secondary prevention) and (migra* or immigrant* or immigration or settler* or incomer* or foreign born or foreign-born or first-generation immigrant or FGI)
