# Supplementary material for: A systematic review of barriers and facilitators for hepatitis B and C screening among migrants in the EU/EEA region
Source: Front Public Health. 2023 Feb 15;11:1118227. doi: 10.3389/fpubh.2023.1118227 (PMC9975596; doi:10.3389/fpubh.2023.1118227)
Supplement: Supplementary material 2 — Study characteristics by design. [file Table_2.DOCX]

Supplementary Material

**A systematic review of barriers and facilitators for hepatitis B and C screening among migrants in the EU/EEA region**

**Chrissy P.B. Moonen^*^, Casper D.J. den Heijer, Nicole H.T.M. Dukers-Muijrers, Ragni van Dreumel, Sabine C.J. Steins, Christian J.P.A. Hoebe.**

*** Correspondence:** Chrissy Pierre Brigitte Moonen: [chrissy.moonen@maastrichtuniversity.nl](mailto:chrissy.moonen@maastrichtuniversity.nl)

**Supplementary Material 2. Study characteristics by design**

| **Reference** | **Country** | **Design** | **Setting** | **Infection(s) of concern** | **Population**  **(sample size)** | **Intervention (and duration)** | **Outcomes** | **Participation rate in screening** |
| --- | --- | --- | --- | --- | --- | --- | --- | --- |
| **Qualitative studies** | | | | | | | | |
| Azadi 2021 | France | Qualitative study | First Reception Center | HBV, HCV and HIV | Randomly selected newly arrived immigrant men (n=25) | Semi-structured in-depth interviews (1 October 2019 to 30 July 2020) | Acceptability of HIV and hepatitis screening and the perception and experience of screening | n/a |
| Cailhol 2020 | France | Qualitative study | Hospital | HBV, HCV and/or HIV | Pakistani migrant men living with hepatitis C/B and/or HIV (including cured patients) (n=13) | Semi-structured interviews, focus groups and ethnographic observations (July to September 2018) | Life-course trajectories and hepatitis /HIV risk factors, individual and social factors influencing behaviors | n/a |
| Cochrane 2016 | United Kingdom | Qualitative study | Community centers | HBV | Somali immigrants (n=30) | Focus groups and semi-structured interviews (February 2014 to July 2014) | Understanding of hepatitis B and response to testing and contact tracing (Awareness, understanding and stigma of HBV, response to public health messages, overcoming barriers to engagement) | n/a |
| Delilovic 2018 | Sweden | Qualitative study | Stockholm County | HIV, hepatitis and tuberculosis | Authority and healthcare professionals involved in the delivery of health examinations (HE)  (n=41) | Focus groups, individual and group interviews (September 2016 to January 2017) | Perceptions and attitudes  among entities involved in the implementation of the HE and health care professionals carrying out the HE | n/a |
| Duracinsky 2020 | France | Qualitative study | Migration offices in three different regions in France (Office Français de l’Immigration et de l’Intégration—OFII) | HBV, HCV, HIV | Legal migrants over 18 years of age (n=34) | Interviews about rapid screening strategy (October 2016 to March 2017) | Knowledge about HIV and  hepatitis B and C and their treatments, attitude towards HIV and hepatitis testing, knowledge  and opinion on rapid testing, and attitude towards discussing sexuality or drug use with health  professionals | n/a |
| Eborall 2020 | United Kingdom | Qualitative study | Community-based settings, interviewees’ workplace, by phone, private research offices | HBV, HCV, HIV, and TB | Members of migrant communities (n=74), healthcare professionals and participants who had experienced the screening and treatment program (n=23). Total of (n=129) participants. | Focus groups and semi-structured interviews (November 2016 to February 2017) | Awareness and perceived risk, views and experiences of combined infection screening, targeted screening and potential stigmatization, receiving test results and treatment | n/a |
| Hamdiui 2018a | The Netherlands | Qualitative study | Community organizations  (community and day care centers, mosques, interest groups  and civil support foundations) located in various cities | HBV | First-generation immigrants (n=9) and second-generation immigrants (n=10) | Semi-structured interviews (duration not mentioned) | Determinants associated with the intention to participate in HBV testing | n/a |
| Hendy 2019 | United Kingdom | Qualitative study | Community hubs (mosques, women’s centers, GP surgeries with high levels of attendance by South Asians) | HBV, HCV | First-generation individuals of 18 years or older from India, Pakistan or Bangladesh (n=53) | 8 focus groups (duration not mentioned) | Sought to develop an inductive theory to explain the  process underlying the readiness to  engage in hepatitis B or C screening. Disease identity and individual identity, the role of social networks and support, and identity misalignment | n/a |
| Kalengayi 2016 | Sweden | Qualitative study | Primary health care centers, Swedish  for Immigrants (SFI) Schools and reception facilities for asylum seekers in four counties | Various infectious diseases | New migrants (n=26) | Interviews (April to June 2013) | Experiences and perceptions about the screening process | n/a |
| Koc 2019 | Belgium | Qualitative aspect in observational study  Longitudinal epidemiological study (protocol for screening) | Unclear where the focus group discussion was held. Screening setting: hospital, in the community and at home | HBV | Focus groups: Delegates of mosques (n=4) and Turkish responsibles (n=2) | See Koc 2020 | See Koc 2020 | n/a |
| Lee 2017 | United Kingdom | Qualitative study | Community, health service, and commissioning level | HBV | Community members and Chinese patients (n=20), clinicians (n=21), health service commissioners (n=9) | In-depth interviews (September 2013 to September 2015) | Determinants of hepatitis B testing and healthcare access  Identify barriers and enablers of hepatitis B testing and healthcare access | n/a |
| Louka 2019 | Greece and the Netherlands | Qualitative study | Greece: the Structure of Welcoming  and Hosting of Refugees  Netherlands: offices of ‘Vluchtelingen-werk’, the tuberculosis center and the Department of Internal Medicine of the UMCG | TB, Scabies, (and preferred expansion for HBV, HCV, HIV) | Refugees (n=61) | Interviews in Greece (May and June of 2018) and in the Netherlands (October 2017 to February 2018) | Demographic data, vaccination status, screening policies  and prevention of infectious diseases | n/a |
| Santilli 2018 | France and Italy | Qualitative study  (ethnograpihic research) | (2) Associations (COMEDE in Paris and SAMIFO in Rome) dealing with medical, psychological and social issues of immigrants applying for a residence permit | HBV | Immigrant adults taken into care (n=30) | Semi-structured interviews (2014 to 2015) | Sociodemographic variables, access to screening, HBV and residence permit. | n/a |
| **(Non) randomized trials** | | | | | | | | |
| Bottero 2015 | France | A single-center randomized controlled trial | Free Clinic | HBV, HCV, and HIV | Persons without healthcare coverage, representing mainly African immigrants  (n=342, 162 in S-arm and 162 in RT-arm) | **Intervention**: Point-of-care rapid testing (RT-arm)  **Control**: Standard serology-based testing (S-arm)  (25 February 2013 to 21 June 2013) | Proportion of participants who became aware of their HIV, HBV, and HCV status and linkage to care when positive | Intervention: 96·9% Control: 63·6% |
| Flanagan 2018 | United Kingdom | (Multicenter, open-label, cluster-) randomized controlled trial | General practices | HBV and HCV | First or second-generation migrants from a high-risk country (aged 18 years or older) patients in primary care  (n=90,250) | **Intervention**: Targeted screening groups:  1) standard (i.e., hospital-based) care and a standard  invitation letter;  2) standard care and an enhanced invitation letter;  3) community care and a standard invitation letter; 4) community care and an enhanced invitation letter·  **Control**: Opportunistic screening group  (31 October 2013 to 4 February 2017) | The proportion of patients/ participants to be screened, that were sent an invitation letter, that attended for screening, that tested positive, that complied with therapy, and the cost-effectiveness | Intervention: 19·5%  Control:  1·7% |
| Ho 2020 | Belgium | Non-randomized trial | Community setting (churches, temples, local public library, massage parlours, and during mandatory integration classes) | HBV | First- or second-generation Asian migrants aged 18 years or older (n=575) | **Intervention**: HBsAg screening by Point-of-care (POC) finger-stick testing  **Control**: Venipuncture and serologic testing for HBsAg  (October 2014 to May 2018) | Screening cost and linkage to care | Intervention: 88·8%  Control: 100% |
| Petroff 2021 | Germany | Non-randomized trial | Primary care setting (private practices) | HCV | Patients (n=622, of which 16% immigrants) | **Intervention**: HCV Point-of-Care testing by use of the Xpert HCV Viral Load Fingerstick  **Control**: Anti-HCV (Cobas) and HCV-RNA (Cobas Amplicor version 2.0, only performed if anti-HCV was positive)  (September 2019 to October 2020) | Diagnostic performance and practicability of the test | Unclear |
| Sequeira-Aymar 2021 | Spain | (Pilot) randomized cluster controlled trial | Primary Care Centers (n=8) in 4 areas of Catalonia | HBV, HCV, HIV TB, Chagas disease, strongyloidiasis and  schistosomiasis | Migrants aged 16 years or older (n=15,780) | **Intervention**: Digital tool for screening recommendation based on individual risk assessment IS-MiHealth  **Control**: Routine care, but were given a training session before the intervention started  (March 2018 to December 2018) | Demographic characteristics, prevalence of HBV, HCV, HIV TB, Chagas disease, strongyloidiasis and schistosomiasis markers, screening performance | Intervention:  HBV: 11.8% HCV: 12.5%  Control:  HBV: 9.2%  HCV: 8.9% |
| Thonon 2021 | France | Stepped wedge randomized trial | 16 centers taking care of and treating migrants; OFII, PASS, non-profit organizations, GP, and gynecologists. | HIV, HBV and HCV | Migrants over 18 years old who have low French proficiency, fluent in a language available in app (study not finished yet) | **Intervention:** Use of the app during consultations with non-French speaking migrants to offer, explain, and carry out the screening test, with the possible help from professional interpreters if necessary  **Control**: Typical processes used by centers for screening migrants (use of phone or in-person interpreters, informal interpreters, or not offering screening tests to migrants because of the language barrier  (has yet to be implemented) | Percentage of screening tests administered, rate of screening proposals, acceptance rate, number of positive cases, frequency of app usage | Study not finished yet (protocol) |
| Kakalou 2021 | Croatia, Italy and Lithuania | Non-randomized trial | Online | Viral hepatitis, HIV, TB and various STIs | Diverse, high-risk groups (i.e. MSM, migrants, PWID, transgender people, sex workers, prisoners) (n=unknown, 1.347/downloads) (6·33% is migrant) | **Intervention**: ‘RiskRadar’ – a web and mobile application aiming to support combination prevention, testing and linkage to care  (6 July 2020 to 31 December 2020) | Number of times used, action taken after RiskRadar use, usefulness, and users’ experience | Unclear |
| **Quantitative descriptive studies** | | | | | | | | |
| Andersen 2020 | Denmark | Cross-sectional study | Hospital | HBV HCV, HIV, and syphilis | Newly settled refugees  (n=160) | General Health Assessment (1 January 2017 to 30 January 2019) | Sociodemographic variables, health status including the prevalence of HBV HCV, HIV, and syphilis markers, migration, and referrals | 81% |
| Bergevin 2021 | France | Single-center retrospective observational study | Hospital | HBV, Schistosomiases, TB, and intestinal parasites | Unaccompanied minors seeking asylum (n=107) | Health Screening at a Pediatric consultation service (1 September 2017 to 30 September 2018) | Prevalence of HBV, Schistosomiases, TB, Intestinal parasitosis markers | Unclear |
| Bil 2018 | The Netherlands | Observational study | TB departments of public health services | HBV, HCV, HIV, and TB | Migrants aged 18 years or older  (n=461) | Integrated HBV, HCV, and HIV screening when attending compulsory TB entry screening  (July 2015 to August 2015) | Prevalence of HBV, HCV and HIV infection | 54% |
| Buonfrate 2018 | Italy | Retrospective observational study | Refugee shelters | HBV, HCV, HIV, Syphilis, LTBI, and strongyloidiasis | Asylum seekers over 14 years of age  (n=462) | Infectious disease screening (April 2014 to June 2015) | Prevalence of viral diseases/infections, bacterial diseases and helminthic infections | 96% |
| Chernet 2018 | Switzerland | Cross-sectional study | Refugee centers | HBV, HCV, HIV, schistosomiasis, helminths/protozoa, malaria, scabies, and syphilis | Asymptomatic Eritrean migrants aged 16 years or older  (n=107) | Infectious disease screening and stool testing (February 2016 to November 2016) | Prevalence of HBV, HCV, HIV, schistosomiasis, helminths/protozoa, malaria, scabies, syphilis, | 80·1% |
| Coenen 2016 | The Netherlands | Multicenter observational design | Outreach location (community centers such as Chinese schools, and churches, Municipal Public Health Service, | HBV | First-generation Chinese migrants (n=4,423) | Disease awareness activities and free HBV testing (2009 to 2013) | HBsAg, HBeAg, and the proportion of treatment indications or follow-up | 15% |
| Colucci 2021 | Italy | Cross-sectional study | ARCA (NGO to provide legal, social and health assistance) | HBV, HCV, HDV and HIV | Migrants (n=362) | Infectious disease screening (March 2019 to February 2020) | Prevalence of HBV, HCV, HDV and HIV markers | 80% |
| Coppola 2015 | Italy | Multicenter prospective study | 4 first-level clinical centers | HBV, HCV and HIV | Undocumented migrants and refugees (n=882) | Infectious disease screening (January 2012 to June 2013) | Prevalence of HBV, HCV and HIV markers | 95·2% |
| Coppola 2017 | Italy | Multicenter prospective study | 6 first-level clinical  centers | HBV, HCV, and HIV | Undocumented immigrants and refugees (n=1,212) | Infectious disease screening (January 2012 to December 2014) | Prevalence of HBV, HCV and HIV markers | 91% |
| Coppola 2020 | Italy | Multicenter prospective study | 7 first-level clinical centers | HBV, HCV and HIV | Immigrants (n=4,125) | Infectious disease screening (January 2012 to June 2018) | Prevalence of HBV, HCV and HIV markers | 93% |
| Cortier 2022 | France | Observational study | Primary Care Unit (PCU) on site of a Primary Reception Center (CPA) | HBV, HCV, HIV, TB, Scabies, fungal skin infections, bacterial skin infections, genitourinary infections, gastrointestinal infections, HSV and HPV infections, syphilis, schistosomiasis, malaria, and shingles | Asylum seekers (only men) (n=728) | Health status screening (January 2018 to March 2018) | Prevalence of HBV, HCV, HIV, TB, Scabies, fungal skin infections, bacterial skin infections, genitourinary infections, gastrointestinal infections, HSV and HPV infections, syphilis, schistosomiasis, and malaria | 77% |
| Crawshaw 2018 | United Kingdom | Cross-sectional study | International Organization for Migration (IOM) Clinics (22) | HBV, HCV, HIV, TB, STDs, and syphilis | Refugees  (n=18,418) | Pre-entry health assessment  (March 2013 to August 2017) | Prevalence of HBV, HCV, HIV, TB, and syphilis markers | 96·3% |
| Cuenca-Goméz 2016 | Spain | Descriptive study | Specialized doctor’s office | HBV, HCV, HDV, and HIV | African immigrants (n=2,518) | Medical history review and complete physical examination, including laboratory and serology tests (October 2004 to June 2015) | Prevalence of HBV, HCV, and HDV markers | Unclear |
| Del Pinto 2018 | Italy | (Monocentric) cross-sectional study | Local reception center | HBV, HCV, HIV, TB, and syphilis | Male refugees from Africa and Asia  (n=93) | Infectious disease screening (July 2014 to December 2014) | Prevalence of HBV, HCV, HIV, TB, and syphilis markers | 100% |
| Donisi 2020 | Italy | Observational retrospective study | Migration Health Unit | HBV, HCV, HIV, TB, and syphilis | Asylum-seekers older than 14 years of age (n=316) | Infectious disease screening (1 January 2015 to 31 December 2015) | Prevalence of HBV, HCV, HIV, TB, and syphilis markers | 99·7% |
| Eonomopoulou 2017 | Greece | Cross-sectional study | Clinical assessment upon arrival at the holding center | HBV, HCV, and HIV | Migrants at the Greek-Turkish border (n=6,899) | Physical conditions and infectious disease screening (1 March 2011 to 31 July 2011) | Demographical data, prevalence of HBV, HCV, HIV, and TB markers, and physical conditions and symptoms | Unclear |
| Evlampidou 2016 | United Kingdom | Cross-sectional survey study | NHS patient demographic data and HBV laboratory surveillance data. Online survey for GP practices. | HBV | Of 82561 individuals in this study population, 9627 (12%) had evidence of an HBV test.  And GP's (n=19) | Linkage of NHS patient demographic data and HBV laboratory surveillance data and an online GP survey was undertaken. | Hepatitis B testing coverage and infection, and  GP views, practices and barriers to HBV testing | 12% HBV testing coverage |
| Fiore 2021 | Italy | Cross-sectional (survey) study | Immigration centers | HBV, HCV, HIV, TB, Syphilis, molluscum contagiosum, and HPV (STD's) | Refugees (n=61) | Infectious disease screening (November 2019 to December 2019) | Prevalence of HBV, HCV, HIV, TB, Syphilis, molluscum contagiosum, and HPV markers | HCV: 76·5% HBV: 67·9% |
| Hamdiui 2018b | The Netherlands | Cross-sectional survey study (distributed by the use of respondent-driven sampling) | Online and offline (community venues, such as community centers, daycare  centers, mosques, interest groups, and civil support  foundations) | HBV | First- and second/third-generation Moroccan-Dutch immigrants (n=379) | Offline and online questionnaires (November 2016 to February 2017) | Determinants that have the greatest impact on (1) the intention to request an HBV screening test on one’s own initiative,  and (2) the intention to participate in non-refundable HBV screening at €70,-. | n/a |
| Hamdiui 2021 | The Netherlands | (Offline) cross-sectional survey study using RDS | Community venues (mosques, day care centers in municipalities) | HBV | First and second-generation immigrants (n=295) | Paper-based questionnaires (February 2019 to  November 2019) | Awareness, knowledge and information needs | n/a |
| Hannula 2021 | Norway | Cross-sectional study | Local opioid substitution clinic, outpatient clinics, PWUD day centers, local prisons and refugee centers | HCV | High-risk  Populations (immigrants, prisoners, PWUD) (n=381, of which 52 immigrants) | HCV screening (September 2015 to March 2017) | Prevalence of HCV markers | 100% (all who attended the meetings signed up to the study) |
| Hargreaves 2020 | United Kingdom | Cross-sectional study | UK emergency department | HBV, HCV, HIV, TB/LTBI | Economic migrants, asylum seekers and refugees (n=93) | Infectious disease screening (duration not mentioned) (follow-up duration 2 years, 9-month recruitment period) | Prevalence of HBV, HCV, HIV, TB markers | Unclear |
| Janda 2020 | Germany | Cross-sectional study | Single private pediatric practice | HBV, HIV, TB | Unaccompanied refugee minors (n=890) | Questionnaire and multi-disease screening (January 2016 to December 2017) | Prevalence of TB, HBV, HIV makers and stool and urinary parasites | 96·1% |
| Kelly 2020 | United Kingdom | Cross-sectional study (prospective descriptive study) | Community, faith, and primary care settings | HBV and HCV | First-generation South Asian migrants over 18 (n=219) | Film (intervention) with dried blood spot CVH testing offered immediately afterwards (February 2018 to June 2018) | (1) the feasibility of recruiting South Asian migrants to view an  educational  film on CVH, (2) the effectiveness of the film in promoting testing and increasing knowledge of  CVH, and (3) the methodological issues relevant to scale-up to a randomized controlled trial  (and exposure to CVH (Chronic Viral hepatitis)) | 84% |
| Klok 2021 | The Netherlands | Cross-sectional study | NGO healthcare facility | HBV, HCV, HIV | Undocumented migrants and uninsured legal residence (n=784) | Infectious disease screening (October 2018 to October 2019) | Prevalence of HBV, HCV, and HIV markers, and follow-up and treatment | 32% |
| Kloning 2018 | Germany | Retrospective cross-sectional study | Pediatric practices and collective housing | HAV, HBV, HCV, HIV, syphilis, TB, Helicobacter pylori, amoeba, lamblia, parasites | Unaccompanied refugee minors (n=154) | Medical examination (October 2014 until February  2016) | Prevalence of HAV, HBV, HCV, HIV, syphilis, and TB markers, and results of stool analysis and hematological results | Unclear |
| Koc 2020 | Belgium | Longitudinal epidemiological study | Community setting (Islamic mosques and Turkish organizations), hospital setting and home setting | HBV | Turkish migrants aged 18 years or older (n=1,081) | Educational sessions and HBV Screening (1 September 2017 to 2 May 2019, follow up until 2 November 2019) | Prevalence of HBV infection and associated risk factors | 97% after educational meetings and 100% at home visits |
| Mandel 2019 | Italy | Retrospective cross-sectional study | Infectious Disease Clinic and  Dermatology Unit of Modena | HBV, HCV, HIV, Syphilis | Patients referred to the T&C-IDC and STDs-DU (n=9,154, of which 29% immigrants) | Infectious disease screening (January 2010 to December 2016) | Demographic and laboratory data, linkage to care | Unclear |
| Marquardt 2016 | Germany | A cross-sectional survey (with purposive sampling) | Private outpatient  clinic for internal and tropical medicine | HBV, TB, Helicobacter pylori, Helminthic diseases, Schistosomiasis, Amoebiasis, Lambliasis | Unaccompanied asylum-seeking minors aged 12-18 years (n=102) | Medical check-up and medical check-up visit (8 September 2011 to 3 July 2014) | Sociodemographic characteristics, general health status, health behavior, burden of infections, mental illness burden, non-communicable diseases burden, referrals and medicines prescription | Unclear |
| Marrone 2020 | Italy | Cross-sectional study | Reception centers | HBV, HCV, TB (LTBI) and syphilis | Unaccompanied immigrant minors aged 13-18 (n=879) | Infectious disease screening (January 2013 to January 2019) | Prevalence of HBV, HCV, TB (LTBI) and syphilis markers | Unclear |
| Mazzitelli 2021 | Italy | Retrospective cross-sectional study | Migrant  outpatient clinic | HBV | Migrants residing in centers (7) for refugees and asylum seekers (n=330) | HBV screening (August 2015 to October 2018) | Patient characteristics, prevalence of HBV markers and follow-up | 87·9% |
| Norman 2021 | Spain | Retrospective cross-sectional study | Specialized unit (clinic) | HAV, HBV, HCV, HIV, rubella, measles, mumps, and varicella | Regular migrants, irregular migrants, asylum seekers and refugees (n=468) | Screening of infectious diseases (2018 to 2019) | Prevalence of HAV, HBV, HCV, HIV, rubella, measles, mumps, and varicella markers | HBV: 97·4% HCV: 95·7% |
| Oliván-Gonzalvo 2021 | Spain | Cross-sectional study with retrospective design | Specialized unit in UIMs of the pediatrics and adolescence service of the Social Services Institute of Aragón | HBV, HCV, TB, HIV, and syphilis | African male unaccompanied immigrant minors (n=622) | Health status and infectious disease screening (2005 to 2019) | Prevalence of HBV, HCV, TB, HIV, and syphilis markers and outcomes of complete physical examination | Unclear |
| Pavlopoulou 2017 | Greece | Prospective, cross-sectional study | Migrant outpatient clinic of a tertiary Children’s hospital | HBV, HCV, and TB | Immigrant and refugee children (1-14 years of age) (n=300) | Examination for health certificate within 3 months after arrival  (2010 to 2013) | Demographics, vaccination status, clinical findings, laboratory screening findings and infectious diseases findings (including prevalence of HBV, HCV and TB markers) | Unclear |
| Picchio 2021 | Spain | Prospective cohort study | Community (faith-based and community organizations  in the greater Barcelona area) | HBV | West  African migrant adults (≥ 18 years) (n=274) | Point-of-care testing and simplified diagnostic tools to identify, link to care or vaccinate African migrants during the COVID-19 pandemic (November 2020 to January 2022) | HCV test results, sociodemographic and clinical history, post-test counselling, vaccination, and linkage to a specialist | 100% |
| Prestileo 2022 | Italy | Prospective study | (41) reception centers across the Sicilian provinces | HBV, HCV, HIV, and HDV (if HBV positive) | Irregular African migrants to Sicily (aged 14-41) (n= 2,639) | Structured questionnaire and multi-disease screening (January 2015 to December 2017) | Prevalence of HBV, HCV, and HIV markers, sociodemographic variables, and risk factors | 95·9% |
| Roche 2021 | United Kingdom | Cross-sectional survey study | Primary care | HBV, HCV and HIV | Primary care professionals (n=414) | Pre-piloted questionnaire (October 2017 to January 2018) | Respondent and practice characteristics, Clinical questions for clinical staff, practice policy questions, barriers for migrants accessing healthcare | n/a |
| Rosa-Hezode 2019 | France | Prospective non-interventional  research study | (2) Primary healthcare facilities in the Créteil Access to Care cluster | Focus on HCV, but also tests for HBV and HIV | Adult migrants attending  two primary healthcare facilities in the Créteil Access to  Care cluster (n=3,386) | Free screening for HBV, HCV, and HIV (May 2007 to December 2017) | Prevalence of HBV, HCV, HIV markers, socio-demographic factors, number of patients informed, (lost to) follow-up, linkage to care, and treatment | Unclear |
| Rosenkrands 2020 | Denmark | Cross-sectional survey | Pioneer clinic specifically addressing immigrant patients with multi-morbidity, complex symptoms and adherence  problems | HBV and HCV (and other communicable and non-communicable diseases) | Immigrants with complex health needs (n=408) | Screening questionnaire and blood test (1 January 2014 to 20 November 2017) | Socio-demographic characteristics, migration history including life events,  healthcare use, symptoms and self-reported morbidity | Unclear |
| Sagnelli 2018 | Italy | Multicenter prospective study | 5 first-level clinical centers in southern Italy | HBV, HCV and HIV | Undocumented immigrants and low-income refugees (n=1,727) | Multiple disease screening (January 2012 to 2015) | Prevalence of HBV, HCV and HIV markers, sociodemographic characteristics, risk factors, follow-up, treatment | 85% |
| Salas-Coronas 2018 | Spain | Retrospective cross-sectional study | Tropical Medicine Unit of the Hospital de Poniente | HBV, HCV, HIV, TB syphilis (and stool parasites) | Newly arrived African immigrants (n=523) | Screening of imported diseases (October 2004 to February 2017) | Sociodemographic variables, the prevalence of HBV, HCV, HIV, syphilis (and stool parasites) markers | Unclear |
| Scotto 2019 | Italy | Cohort study | Reception center (CARA) | HBV, HCV, TB, HIV | Newly arrived, asymptomatic Sub-Saharan African and Asian immigrant men (n=238) | Multiple disease screening, interview using a questionnaire (January 2015 to December 2015) | Demographic characteristics and prevalence of HBV, HCV, TB, and HIV markers | 28·2% |
| Serre Delcor 2018 | Spain | Descriptive retrospective population study | Vall d’Hebron-  Drassanes Tropical Medicine and International Health Unit  In Barcelona | HBV, HCV, HIV, TB and syphilis (and endemic infections from tropical areas, including malaria, schistosomiasis,  filariasis, and intestinal parasites) | Asylum seekers who requested a medical exam (with or without symptoms) (n=303) | Multiple disease screening (July 2013 and June 2016) | Demographic characteristics, reason for consultation, prevalence of (infectious and non-communicable diseases), adherence to follow-up and vaccination | 82·5% |
| Tiittala 2018 | Finland | Cross-sectional retrospective register-based  study | Reception center | HBV, HIV, TB, and syphilis | Asylum seekers (n=37,614) | Multi-disease screening based on individual risk assessment (1 January 2015 to 31 December 2016) | Prevalence of eligibility, coverage of screening, timing of screening, prevalence of infections | 60·6% |
| Touloumi 2020 | Greece | (3) cross-sectional (epidemiological surveys) study | Greek medical schools, the MSc  International Medicine—Health Crisis Management of the Medical School of NKUA, and the non-governmental  organizations (NGOs) Doctors of the World Greek delegation and PRAKSIS | HBV, HCV, HIV | 3 adult (≥18 years) populations  (n=7152)  (1) the general population (n=6,006)  (2) Greek Roma, and (n=534)  (3) migrants (n=612) | Hprolipsis: hepatitis and HIV screening program (including surveys and awareness activities)  (May 2013 to June 2016) | Prevalence of HBV, HCV, and HIV; infectious disease knowledge level; population-specific  awareness actions; individual counselling and referral | 87·7% |
| Williams 2020 | United Kingdom | Cross-sectional study | (2) Pediatric infectious diseases  clinics | HBV, HCV, HIV, TB, and schistosomiasis | Unaccompanied asylum-seeking children  and young people aged 18 years or under (n= 252) | Multi-disease screening (January 2016 to  December 2018) | Demographic characteristics and prevalence of HBV, HCV, HIV, TB, and schistosomiasis markers | 84% |
| Zampino 2018 | Italy | Descriptive study | Primary Care Physicians (15 GPs) and Hospital Liver Units (3) | HBV | Patients (n=30), 10% migrant (3/30) | Cooperation strategy between Primary Care Physicians and Hospital Liver Units on HBV Care (February 2016 to February 2017) | Enrolment, referral to hospital liver units, specialist follow-up, and data of HBsAg patients enrolled | 10·6% |
| **Mixed-method studies** | | | | | | | | |
| Padovese 2020 | Malta | Quantitative and qualitative research within a single center | Sexual health clinic | Infectious diseases including Hepatitis B and C, HIV and sexually transmitted infections | Non-European migrants (n=143) | Questionnaire and interviews (January to June 2019) | Prevalence and knowledge, attitude and practice (KAP) | n/a |
